# Peri-event plasma PCSK9 and hsCRP after an acute myocardial infarction correlate with early deterioration of left ventricular ejection fraction: a cohort study

**DOI:** 10.1186/s12944-022-01672-4

**Published:** 2022-07-21

**Authors:** Lina S. Silva-Bermúdez, Andrea Vargas-Villanueva, Carlos A. Sánchez-Vallejo, Ana C. Palacio, Andrés F. Buitrago, Carlos O. Mendivil

**Affiliations:** 1 Universidad de los Andes, School of Medicine, Carrera 7 No 116-05, Of 413, Bogotá, 110111 Colombia; 2 Critical Care and Intensive Medicine Department, Fundación Santa Fe de Bogotá, Bogotá, Colombia; 3 Cardiology Section, Internal Medicine Department, Fundación Santa Fe de Bogotá, Bogotá, Colombia; 4 Endocrinology Section, Internal Medicine Department, Fundación Santa Fe de Bogotá, Bogotá, Colombia

**Keywords:** Cardiovascular disease, Ischemic heart disease, Heart failure, Hispanic, Latino

## Abstract

**Background:**

It is important to identify patients at increased risk of worsening of left ventricular ejection fraction (LVEF) after a myocardial infarction (MI). We aimed to identify the association of various potential biomarkers with LVEF impairment after an MI in South American patients.

**Methods:**

We studied adult patients admitted to a University Hospital and diagnosed with an acute MI. Plasma concentrations of high-sensitivity C-reactive protein (hsCRP), proprotein convertase subtilisin/kexin type 9 (PCSK9), N-terminal prohormone of brain natriuretic peptide (NT-proBNP) and heart-type fatty-acid-binding protein (FABP3) were determined in samples drawn shortly after the event. Participants had a follow-up visit at least 45 days after the event. The primary endpoint was defined as any decline in LVEF at follow-up relative to baseline.

**Results:**

The study included 106 patients (77.4% men, 22.6% women), mean age was 64.1, mean baseline LVEF was 56.6, 19% had a prior MI. We obtained a follow-up evaluation in 100 (94.4%) of participants, mean follow-up time was 163 days. There was a significant correlation between baseline PCSK9 and hsCRP (r = 0.39, *p* < 0.001). Baseline hsCRP concentrations were higher in patients who developed the endpoint than in those who did not (32.1 versus 21.2 mg/L, *p* = 0.066). After multivariate adjustment, baseline PCSK9, male sex and age were significantly associated with impairment in LVEF. The absolute change in LVEF was inversely correlated with baseline hsCRP (standardized coefficient = − 0.246, *p* = 0.004).

**Conclusion:**

High plasma levels of PCSK9 and hsCRP were associated with early decreases in LVEF after an MI in Latin American patients.

## Background

Cardiovascular diseases (CVD) are the leading mortality cause worldwide, resulting in an estimated 22 million deaths by 2030 [1]. Coronary heart disease represents the largest proportion of CVD [2]. After an initial event of myocardial infarction (MI), heart failure (HF) is the most frequent complication [3], with an in-hospital incidence of 14-36% [4] and a one-year incidence of up to 32% [5]. The absence of left ventricular ejection fraction (LVEF) recovery after an MI predicts adverse morbidity and mortality outcomes [6]. Patients who develop heart failure have an average five-year survival lower than patients with bowel, ovarian or breast cancer [7]. Even when the decline is as small as 5%, failure to restore LVEF has been associated with a higher risk of death in post-MI patients [8, 9]. The decline in LVEF also influences the need for continued medications after an MI, as is the case for beta-blockers, RAAS inhibitors and mineralocorticoid antagonists [10, 11].

After the inflammatory process that characterizes a MI, myocardial fibroblasts contribute to early scar deposition and pathological remodeling of ventricular fibers. The renin-angiotensin-aldosterone (RAA) and sympathetic nervous systems also contribute to wall thinning, cardiomyocyte loss and changes in ventricular geometry that result in ventricular dysfunction [12, 13]. Over time, this neurohumoral activation causes destruction of cardiomyocytes in other non-ischemic areas, resulting in progressive loss of cardiac function [14]. Predicting which patients will develop such complication over the months after an MI can be challenging [15]. Hence, there is an unmet need for biomarkers of future ventricular dysfunction, which may themselves be therapeutic targets [16].

Several biomarkers have proven useful in the diagnosis and follow-up of HF or its precursor conditions. For instance, plasma levels of N-terminal pro-brain natriuretic peptide (NT-proBNP) are a relevant decision-making tool in HF diagnosis and follow-up [17]. Heart-type fatty acid-binding protein (FABP3) and troponin are proteins released into circulation after myocardial injury that have been used as markers of ongoing myocardial damage and thus, later cardiac remodeling [17]. Furthermore, high levels of peri-event plasma inflammatory biomarkers predict the development of incident HF in patients with MI [18]. Among the many available inflammatory biomarkers, high sensitivity C-reactive Protein (hsCRP) has been associated with more severe features of HF with preserved ejection fraction (HFpEF) [19]. Other markers that focus on low-density lipoprotein metabolism may also be relevant, like Proprotein Convertase Subtilisin/Kexin Type 9 (PCSK9). PCSK9 may serve not only as a biomarker of coronary atherosclerosis [20, 21], but also for the worsening of HF [22]. Higher levels of PCSK9 after an ST-elevation Myocardial Infarction (STEMI), have been associated with lower post follow-up LVEF in patients with reduced ventricular function at baseline [23].

The development of biomarkers for the decline in ventricular function after an MI would be a useful addition in the identification of patients at higher risk of adverse outcomes [24]. With this background, the aim of our study was to evaluate the association between plasma concentrations of candidate biomarkers right after an MI and subsequent deterioration of LVEF, in South American patients.

## Methods

### Study design and participants

Sample size was calculated employing the expression for prospective observational studies [25], with a minimum expected incidence of LVEF reduction among non-exposed (baseline PCSK9 in the lowest quartile) of 30% [26], a relative risk of LVEF impairment of 2.2 among participants in the highest quartile of PCSK9, at a 0.05 significance level and 80% power. The obtained sample size was 104 participants.

We recruited between January 2017 and October 2019 a total of 106 patients (24 women and 82 men), who were admitted to University Hospital Fundación Santa Fe de Bogotá and diagnosed with an acute MI according to the “Third Universal Definition of Myocardial Infarction” [27]. Inclusion criteria were age 18 years or older, admission with either an ST-segment elevation myocardial infarction (STEMI) or a non-ST elevation myocardial infarction (NSTEMI), absence of a prior diagnosis of heart failure, and willingness to take part in the study. All consecutive patients who consented to participate, were included in the study. STEMI was defined as symptoms of myocardial ischemia plus a new ST-segment elevation ≥ 0.1 mV in two or more contiguous leads, except in precordial lead V2, in which different cutoffs were used according to sex and age. For women, an ST segment elevation > 0.15 mV was considered positive. Men under 40 years required ST segment elevation > 0.25 mV, while men over 40 years were considered positive if they had an ST segment elevation > 0.2 mV. Another EKG pattern considered positive was new or presumably new left bundle branch block. NSTEMI was defined as the presence of ischemic symptoms without the aforementioned electrocardiographic changes and increasing or decreasing levels of high-sensitivity T-Troponin (hs-cTnT) with at least one value above the 99th percentile upper reference limit.

After enrolment in the study, relevant clinical and sociodemographic information was collected, including smoking status, medical history of diabetes, hypertension, dyslipidemia, any history of cancer, any prior myocardial infarctions, as well as current statin and antihypertensive therapy. A venous blood sample was drawn in EDTA tubes. Plasma was promptly separated, added a preserving cocktail (benzamidine, phenylmethylsulphonyl fluoride and gentamicin), aliquoted, and stored at − 80 °C for later analyses. Participants underwent transthoracic echocardiography and coronary angiography at the index hospitalization, from which LVEF, percent maximal coronary obstruction and number of obstructed coronary vessels were obtained. In all participants, percutaneous coronary intervention was performed promptly according to institutional guidelines with a delay < 60 minutes for patients with STEMI, < 2 hours for high-risk patients with NSTEMI and < 24 hours for all other patients with NSTEMI. After acute care for their MI, all patients received state-of-the-art, evidence-based therapy for coronary heart disease including high-intensity statin, RAAS inhibition, dual antiplatelet therapy and beta-adrenergic blockade.

Participants had a follow-up visit at least 45 days after the initial blood draw. In this visit, a second sample was collected, a new echocardiogram was performed, and the occurrence of new major adverse cardiovascular events (MACE) was documented, including cardiovascular death, non-fatal ischemic cardiovascular events, coronary revascularization, need for re-intervention or hospitalization due to unstable angina.

### Measurements

In both plasma samples, we determined using sensitive immunoassays the plasma levels of hsCRP (R&D Systems Quantikine® ELISA Human C-Reactive Protein/CRP Immunoassay, CAT#DCRP00), PCSK9 (R&D Systems Quantikine® ELISA Human Proprotein Convertase 9/PCSK9 Immunoassay, CAT #DPC900), NT-proBNP (Abnova NTproBNP ELISA, CAT # KA3099) and FABP3 (R&D Systems DuoSet® Human FABP3 ELISA, CAT#DY1678). Plasma lipids were measured using conventional colorimetric assays (Biosystems, Costa Brava, Spain). Glycated hemoglobin A1c (HbA1c) was determined using a National Glycohemoglobin Standardization Program (NGSP)-certified boronate affinity technique (NycoCardTM Reader II, Alere Technologies, Norway), or an HPLC-based, NGSP-certified technique (Premier Hb9210, Trinity Biotech, Ireland).

Transthoracic echocardiography was performed by an institutional cardiologist/echocardiographist who was blinded to laboratory results, using a Siemens SC2000® ultrasound machine. The left ventricular ejection fraction (LVEF) was calculated following the biplane Simpson’s method [28]. In order to reduce the inter-observer variability, the same person performed the exam at the two visits. The coronary angiographies were performed by a trained cardiologist, who recorded the number of obstructed coronary arteries and maximum degree of coronary occlusion. The primary endpoint was a categorical variable defined as a decline in LVEF at the follow-up visit, relative to the baseline value.

### Statistical analysis

Means of baseline plasma concentrations of biomarkers were compared using t-tests for normally-distributed and Mann-Whitney’s U-test for non-normally distributed variables. The association of demographic and clinical variables, and of baseline biomarkers with the primary endpoint was assessed using multivariate logistic regression analysis. Biomarkers were introduced in the models in quartiles, and all ORs reported in tables are for Q4 relative to Q1. Adjustment was performed in three models. Model 1 included sex, age and basal LVEF as predictors, model 2 included all variables in model 1 plus number of affected coronary vessels and percent maximal coronary occlusion, model 3 (fully adjusted) included all variables in model 2 plus change (follow-up - baseline) in plasma concentrations of the biomarker being examined. The association between the same set of predictors and the absolute numeric change in LVEF was assessed using multivariate linear regression. All tests were done at a 0.05 significance level and all reported *p*-values are 2-sided. Statistical analyses were performed in SPSS statistics, version 23 (SPSS, Cary, NC, USA).

### Ethical considerations

The Internal Review Board (Comité de Ética) of Fundación Santa Fe de Bogotá approved the study according to minute CCEI-6070-2016 of September 19, 2016. We complied with all scientific, technical and administrative norms for health research dictated by Resolution 8430 of 1993 of the Colombian Ministry of Health and with the principles stated by the Declaration of Helsinki. All study subjects underwent an informed consent procedure and provided informed consent.

## Results

### Characteristics of the study sample

The study included 106 adults (77.4% men, 22.6% women), 30 to 95 years old (mean age 64.1). The mean time elapsed between the onset of acute coronary symptoms and the collection of blood for baseline measurements was 50.9 hours (Table 1). Nineteen percent of study participants had a prior myocardial infarction. Mean BMI was 26.7 Kg/m^2^, the prevalence of overweight and obesity were respectively 39.6 and 20.8%. A third of study participants were on statin therapy, while approximately one half took antihypertensive medication. Just 10.4% of participants were current smokers. Even though only 27.4% of participants had a formal previous diagnosis of diabetes, mean HbA1c was in the diabetic range (6.7%). Of note, mean LDL and non-HDL cholesterol levels were not remarkably high (100 and 139 mg/dL [2.6 and 3.6 mmol/L], respectively). In accordance with the study design, baseline troponin (mean 3489 ng/L) and hsCRP (mean 25.5 mg/L) were extremely elevated. Baseline LVEF was on average 56.6%. Out of the 106 patients evaluated at baseline, we obtained a follow-up evaluation in 100 of them, for a 5.6% loss-to-follow-up rate. The average time between the first and second study visits was 163 days. The mean GRACE score of study participants was 103 +/− 25, corresponding to a mean 6-month predicted mortality of 6.0 +/− 5.0%, with higher scores among female participants. The Spearman correlation coefficient between baseline PCSK9 and baseline LVEF − 0.15, and it was not statistically significant (*p* = 0.13).

**Table 1:** Baseline characteristics of study participants

	Women	Men	Total
*n*=	24 (22.6%)	82 (77.4%)	106
Age	68 +/− 13	63 +/− 11.9	64.1 +/− 12.3
Time between onset of symptoms and sample (hours)	52.7 +/− 34.3	50.3 +/− 33.7	50.9 +/− 33.7
Body-mass index (Kg/m2)	25.5 +/− 5	27 +/− 4	26.7 +/− 4.3
Systolic blood pressure (mmHg)	127 +/− 24.4	133.8 +/− 25.2	132.2 +/− 25.1
Diastolic blood pressure (mmHg)	77.9 +/− 12.5	81 +/− 17.6	80.3 +/− 16.6
Heart rate (bpm)	82.4 +/− 18.1	75.6 +/− 20.5	77.1 +/− 20.1
Baseline troponin (ng/L)	3797.2 +/− 12,886	3397 +/− 10,609.3	3489.4 +/− 11,110.3
Glycated hemoglobin A1c (%)	7.0 +/− 1.7	6.6 +/− 1.3	6.7 +/− 1.4
Total cholesterol (mg/dL)	149.2 +/− 45.3	158.5 +/− 52.5	156.4 +/− 50.9
mmol/L	3.84 +/− 1.17	4.10 +/− 1.36	4.05 +/− 1.32
HDL cholesterol (mg/dL)	30.7 +/− 17.8	27.3 +/− 9.6	28.1 +/− 11.9
mmol/L	0.79 +/− 0.46	0.71 +/− 0.25	0.73 +/− 0.31
LDL cholesterol (mg/dL)	96.2 +/− 36.1	101.9 +/− 53.5	100.6 +/− 50.0
mmol/L	2.49 +/− 0.93	2.64 +/− 1.39	2.61 +/− 1.30
Triglycerides (mg/dL)	111.5 +/− 47.0	146.8 +/− 99.1	138.8 +/− 91.0
mmol/L	2.89 +/− 1.21	3.80 +/− 2.56	3.60 +/− 2.36
non-HDL cholesterol (mg/dL)	118.5 +/− 37.0	131.2 +/− 53.3	128.3 +/− 50.2
mmol/L	3.07 +/− 0.96	3.40 +/− 1.38	3.32 +/− 1.30
hsCRP (mg/L)	22.5 +/− 29.6	26.4 +/− 24.2	25.5 +/− 25.5
Baseline plasma PCSK9 (ng/mL)	409.2 +/− 236.5	392.4 +/− 266.2	396.2 +/− 258.8
Baseline plasma NTproBNP (ng/mL)	162.5 +/− 100.4	315.2 +/− 603.9	280.6 +/− 536.3
Baseline plasma FABP3 (ng/mL)	6.3 +/− 8.6	22.3 +/− 61.4	18.6 +/− 54.5
Baseline LVEF (%)	54.0 +/− 10.7	57.4 +/− 8.5	56.6 +/− 9.1
Highest obstruction in coronary vessels (%)	59.0 +/− 40.9	79.1 +/− 31.2	74.8 +/− 34.3
Diabetes (%)	33.3%	25.6%	27.4%
Hypertension (%)	58.3%	54.9%	55.7%
Dyslipidemia (%)	45.8%	53.7%	51.9%
Prior myocardial infarction (%)	13.0%	20.7%	19.0%
History of cancer (%)	25.0%	11.0%	14.2%
Number of affected vessels (%)			
0	20.0%	5.3%	8.4%
1	45.0%	20.0%	25.3%
2	15.0%	36.0%	31.6%
3	0.0%	22.7%	17.9%
4	20.0%	13.3%	14.7%
5	0.0%	2.7%	2.1%
Current use of statins (%)	41.7%	28.0%	31.1%
Current use of antihypertensives (%)	54.2%	47.6%	49.1%
Smoking (%)	4.2%	12.2%	10.4%
GRACE score	111 (27)	101 (23)	103 (25)
Mortality predicted by GRACE score	8.1 (7.2)	5.4 (4.0)	6.0 (5.0)

Data are means +/− SD unless indicated otherwise. *LVEF* Left ventricular ejection fraction, *hsCRP* High-sensitivity C-reactive protein, *PCSK9* Proprotein convertase subtilisin kexin type 9, *NT-proBNP* N-terminal prohormone of brain natriuretic peptide, *FABP3* Heart-type fatty acid binding protein

### Biomarker concentrations according to LVEF decline

**Fig. 1:**
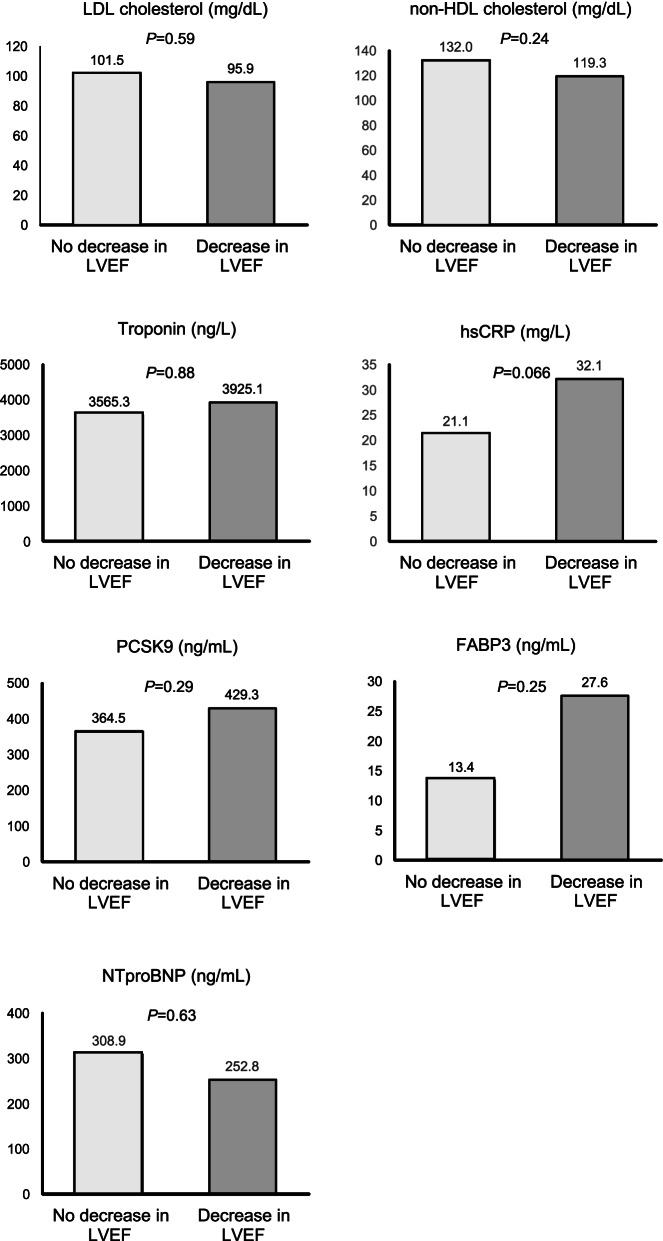
Baseline concentrations of biomarkers in participants according to whether their left ventricular ejection fraction declined or not over the study follow-up

The endpoint of reduction in LVEF occurred in 35 participants. Compared to participants who maintained or increased their LVEF at follow-up, those with the endpoint had numerically higher mean values of baseline plasma FABP3 (27.6 versus 13.4 ng/mL), PCSK9 (429 versus 364.5 ng/mL) and troponin (3925 versus 3565 ng/L), but these differences did not reach statistical significance. However, the difference in baseline hsCRP between these two groups (32.1 versus 21.2 mg/L) was close to reaching statistical significance (*p* = 0.066) (Fig. 1). Unexpectedly, NTpro-BNP was higher in participants who did not have impairments in LVEF over follow-up, albeit this difference was also non-significant. We found linear correlations between baseline plasma concentrations of PCSK9 and hsCRP (r = 0.39, *p* < 0.001), PCSK9 and non-HDL cholesterol (r = − 0.26, *p* = 0.008) as well as between NTpro-BNP and non-HDL cholesterol (r = 0.35, *p* < 0.001).

### Predictors of LVEF impairment

Age was the variable most robustly associated with LVEF impairment during follow-up. After adjustment for baseline LVEF and variables reflecting the severity of atherosclerotic affectation (number of obstructed coronary vessels and percent maximal coronary obstruction), women had 65% lower risk of LVEF decline (Table 2). In the same model, each additional year of age was associated with a 7% increase in the odds of any LVEF reduction. Interestingly, a higher LVEF at baseline was also associated with the probability of reductions after follow-up, each additional 1% of baseline LVEF was associated with 16% higher odds. When comparing extreme quartiles of baseline biomarkers, high concentrations of hsCRP were strongly and positively associated with the risk of LVEF reduction, albeit the association lost significance after adjustment for atherosclerosis extent in model 2 (Table 2). Meanwhile, baseline PCSK9 was positively associated with the endpoint, and this association actually became significant only after full adjustment in model 3. On the other hand, baseline troponin, NT-proBNP and FABP3 were not associated with impairment in LVEF.

**Table 2:** Predictors of reduced left ventricular ejection fraction during study follow-up

	Univariate	Model 1	Model 2	Model 3
Female sex	0.31 (0.01-1.01)	0.23 (0.06-0.95)	0.35 (0.07-1.70)	–
Age	1.04 (1.00-1.08)	1.06 (1.01-1.11)	1.07 (1.01-1.13)	–
Basal LVEF	1.16 (1.08-1.25)	1.16 (1.08-1.25)	1.16 (1.07-1.25)	–
Basal hsCRP (Q4 vs Q1)	2.11 (0.68-6.51)	4.74 (1.03-21.8)	3.52 (0.73-17.1)	5.30 (0.04-659)
Basal PCSK9 (Q4 vs Q1)	0.85 (0.28-2.61)	1.88 (0.40-8.83)	2.63 (0.47-14.7)	17.1 (1.48-198)
Basal NT-proBNP (Q4 vs Q1)	1.26 (0.36-4.42)	0.84 (0.19-3.80)	0.83 (0.16-4.25)	0.63 (0.11-3.77)
Basal FABP3 (Q4 vs Q1)	1.45 (0.44-4.71)	2.33 (0.55-9.94)	1.82 (0.34-9.81)	2.67 (0.38-18.5)

Data are OR (95% CI). For biomarkers, the lowest quartile of plasma concentration was used as reference group. *LVEF* Left ventricular ejection fraction, *hsCRP* High-sensitivity C-reactive protein, *PCSK9* Proprotein convertase subtilisin kexin 9, *NT-proBNP* N-terminal prohormone of brain natriuretic peptide, *FABP3* Heart-type fatty acid binding protein. Model 1 includes sex, age and baseline LVEF as independent variables. Model 2 includes all variables in Model 1 plus number of obstructed coronary vessels and percent maximal coronary obstruction. Model 3 includes all variables in model 2 plus change in the respective biomarker (final-basal plasma concentration)

When the absolute change in LVEF was the dependent variable in a multivariate linear regression model that included sex, age, baseline LVEF and basal levels of all biomarkers, the independent variables with a significant association were age (standardized beta = − 0.194, *p* = 0.013), baseline LVEF (standardized beta = − 0.657, *p* < 0.001) and hsCRP (standardized beta = − 0.246, *p* = 0.004).

## Discussion

In this study of South American patients with an acute MI, we identified factors associated with impairment of LVEF, including clinical characteristics and plasma biomarkers reflecting different aspects of disease pathophysiology. Our results showed that male sex and increasing age were predictors of decreases in LVEF. In multivariate-adjusted models, baseline plasma concentrations of PCSK9 were significantly and strongly associated with subsequent reduction of LVEF, but baseline markers of myocardial tissue damage (troponin and FABP), or myocardial stress (NT-proBNP) were not. On the other hand, the numeric change in LVEF was inversely correlated with concentrations of hsCRP, a marker of systemic inflammation. The absence of a correlation between baseline PCSK9 and baseline LVEF indicates that PCSK9 was a predictor of future deterioration of LVEF over the months after the event, and that this effect was not due to a correlation that existed between PCSK9 and LVEF prior to the myocardial infarction.

Few studies have investigated post-MI LVEF impairment specifically in Latin American patients. Comparative analyses have found that HF with reduced ejection fraction (HFrEF) is more prevalent in this ethnic group than HFpEF [29]. Additionally, LVEF impairment is more frequently associated with ischemic etiologies among patients of Latino ethnicity [29]. Therefore, the identification of predictors of LVEF deterioration after an acute coronary event in this population is of high relevance. Plasma NT-proBNP correlated inversely with LVEF and predicted the development of new HF in a cohort that included Hispanic patients [30]. Our results, however, suggest that this observation may not apply in the context of a recent MI. In a cross-sectional study in Argentina, De Abreu et al. studied the correlation of various plasma biomarkers with acute LVEF in patients with STEMI [31]. There was a significant inverse correlation between NT-proBNP and acute LVEF. However, the prognostic value of the biomarkers for later changes in LVEF was not assessed.

Our finding of an association between baseline PCSK9 and the study endpoint after multivariate adjustment was interesting. Prior research had demonstrated that PCSK9 may behave as an acute phase reactant in acute coronary syndromes [21], and that its synthesis and secretion may be stimulated by hsCRP in vitro [32]. Meanwhile, studies of the prospective association of plasma PCSK9 with a first coronary event in the general population have yielded conflicting results [33–36]. In a recent Spanish study using magnetic cardiac resonance imaging in 40 patients with MI, post-reperfusion plasma PCSK9 was independently and inversely correlated with LVEF at the end of follow-up (6 months), even after adjustment for infarct size and other relevant covariates [23]. Our results also suggest that peri-event PCSK9 concentrations may be associated with increased risk of subsequent loss of ventricular function in some patient populations, and that PCSK9 inhibition with antibodies or gene-silencing interventions may constitute an interesting target for the preservation of LVEF in patients with MI.

In this study, baseline hsCRP correlated independently and negatively with follow-up LVEF. This finding is not restricted to a decrease in LVEF below a pre-established threshold, but is more of a reflection of the association of this biomarker with the absolute change in ejection fraction. These results are consistent with Reinstadler et al. [37], who evaluated the univariate association between peri-event hsCRP following an acute STEMI and left ventricular function measured by cardiovascular magnetic resonance. Plasma hsCRP correlated negatively with LVEF both at baseline and one year later. Nevertheless, a recent study in Croatia found similar baseline hsCRP concentrations between patients who developed reductions of LVEF below 50% one year after a STEMI and those who did not [38]. In addition, the phase II VCU-ART and VCU-ART2 trials using anakinra (a IL-1 receptor blocker) showed a reduction in CRP levels and a reduced incidence of heart failure after 3 months in STEMI patients [39]. Using a design similar to ours, Lakhani et al. explored the utility of several biomarkers for cardiovascular prognosis after an acute MI. The results showed that lower IL-10 during the first 24 hours after the event was associated with lower LVEF two months later [40]. Since IL-10 is considered an anti-inflammatory cytokine [41], this agrees with our results in suggesting that a greater inflammatory response during an MI correlates with more affectation of ventricular function over the following months.

### Strengths and limitations

Strengths of the study include its prospective nature, the specific ethnicity of the patient population, the measurement of multiple biomarkers representing different physiological alterations in acute MI and the low rate of loss to follow-up. Each biomarker was measured in the same centralized laboratory following an identical methodology for all participants.

The main limitations of the study include its limited power due to its small sample size (which may have resulted wide confidence intervals for the associations observed), and the fact that the association of predictors with LVEF reduction was assessed only in the timeframe of a few months. We do not know whether the identified predictors have the same relevance over a longer term. There was some patient heterogeneity as we included patients with both STEMI and NSTEMI, and atherosclerosis severity was not assessed with the SYNTAX score. Also, we did not adjust for antihypertensive therapy, and did not assess the microvascular inflammation status of study participants. We did not have repeated measurements of the analyzed biomarkers, which would help better delineate the relationship between the time course of their concentrations and the evolution of LVEF. Future studies aimed at confirming these interesting findings must include larger numbers of patients with MI and follow them for a more prolonged time.

## Conclusions

In summary, this study identified key predictors of early loss of left ventricular function after an acute MI in Latin American patients. Sex, age, systemic inflammation (as reflected by hsCRP) and baseline concentrations of PCSK9 were important predictors of LVEF worsening. These results help characterize patients who are at increased risk of systolic ventricular dysfunction after an MI, particularly patients of Latino ethnicity.

## Data Availability

The datasets used and/or analysed during the current study are available from the corresponding author on reasonable request.

## Abbreviations

CVD: Cardiovascular diseases
FABP3: Heart-type fatty acid-binding protein
HDL: High-density lipoproteins
HF: Heart failure
HFpEF: Heart failure with preserved ejection fraction
hs-cTnT: High-sensitivity T-Troponin
hsCRP: High sensitivity C-reactive Protein
LDL: Low-density lipoproteins
LVEF: Left ventricular ejection fraction
MACE: Major adverse cardiovascular events
MI: Myocardial infarction
NSTEMI: Non-ST elevation myocardial infarction
NT-proBNP: N-terminal pro-brain natriuretic peptide
PCSK9: Proprotein Convertase Subtilisin/Kexin Type 9
RAAS: Renin-angiotensin-aldosterone system
